# 

**Published:** 1884-01

**Authors:** 


					﻿Vol. IV.
JANUARY, 1884.
NO. 1.
JPHE
gOMlEDPATHlC puY^lClAJfl,
A MONTHLY JOURNAL OF MEDICAL SCIENCE.0
u Seek the Truth: come whence it may, cost what it will."
ZE.CT. LEK, JVE. ZD.,
EDITOR.
CONTENTS:
(See inside of Cover.)
PHILADELPHIA:
No. 2109 CHESTNUT STREET.
3ST E 'W "ST O » -K,:
A. L. CHATTERTON, PUBLISHING COMPANY, PUBLISHER’S AGENTS.
X.O3XT33O2TS
ALFRED HEATH & CO., Sole Agents fob Great Britain,
114 Ebury Street, Eaton Square.
Yearly Subscription, $2,50, invariably in advance.
Entered at the Philadelphia Post-Office as Second-Class Hail Matter.
				

